# The promising novel biomarkers and candidate small molecule drugs in kidney renal clear cell carcinoma: Evidence from bioinformatics analysis of high‐throughput data

**DOI:** 10.1002/mgg3.607

**Published:** 2019-02-21

**Authors:** Bo Zhang, Qiong Wu, Ziheng Wang, Ran Xu, Xinyi Hu, Yidan Sun, Qiuhong Wang, Fei Ju, Shiqi Ren, Chenlin Zhang, Lin Qin, Qianqian Ma, You Lang Zhou

**Affiliations:** ^1^ Medical School of Nantong University Nantong P.R. China; ^2^ The Hand Surgery Research Center, Department of Hand Surgery Affiliated Hospital of Nantong University Nantong P.R. China; ^3^ Department of Medicine Nantong University Xinling college Nantong P.R. China; ^4^ Department of Oncology First Teaching Hospital of Tianjin University of Traditional Chinese Medicine Tianjin P.R. China; ^5^ Department of Spine Chinese medicine hospital Wuxi P.R. China; ^6^ Department of Urology The First people′s Hospital of Taicang City, Taicang Affiliated Hospital of Soochow University Suzhou P.R. China; ^7^ Emergency office Wuxi Center for disease control and prevention Wuxi P.R. China

**Keywords:** bioinformatics analysis, candidate small molecules, kidney renal clear cell carcinoma, novel biomarkers

## Abstract

**Background:**

Kidney renal clear cell carcinoma (KIRC) is the most common subtype of renal tumor. However, the molecular mechanisms of KIRC pathogenesis remain little known. The purpose of our study was to identify potential key genes related to the occurrence and prognosis of KIRC, which could serve as novel diagnostic and prognostic biomarkers for KIRC.

**Methods:**

Three gene expression profiles from gene expression omnibus database were integrated to identify differential expressed genes (DEGs) using limma package. Enrichment analysis and PPI construction for these DEGs were performed by bioinformatics tools. We used Gene Expression Profiling Interactive Analysis (GEPIA) database to further analyze the expression and prognostic values of hub genes.

The GEPIA database was used to further validate the bioinformatics results. The Connectivity Map was used to identify candidate small molecules that could reverse the gene expression of KIRC.

**Results:**

A total of 503 DEGs were obtained. The PPI network with 417 nodes and 1912 interactions was constructed. Go and KEGG pathway analysis revealed that these DEGs were most significantly enriched in excretion and valine, leucine, and isoleucine degradation, respectively. Six DEGs with high degree of connectivity (*ACAA1, ACADSB, ALDH6A1, AUH, HADH,* *and PCCA*) were selected as hub genes, which significantly associated with worse survival of patients. Finally, we identified the top 20 most significant small molecules and pipemidic acid was the most promising small molecule to reverse the KIRC gene expression.

**Conclusions:**

This study first uncovered six key genes in KIRC which contributed to improving our understanding of the molecular mechanisms of KIRC pathogenesis. *ACAA1, ACADSB, ALDH6A1, AUH, HADH,* *and PCCA* could serve as the promising novel biomarkers for KIRC diagnosis, prognosis, and treatment.

## 1 INTRODUCTION

**Figure 1:**
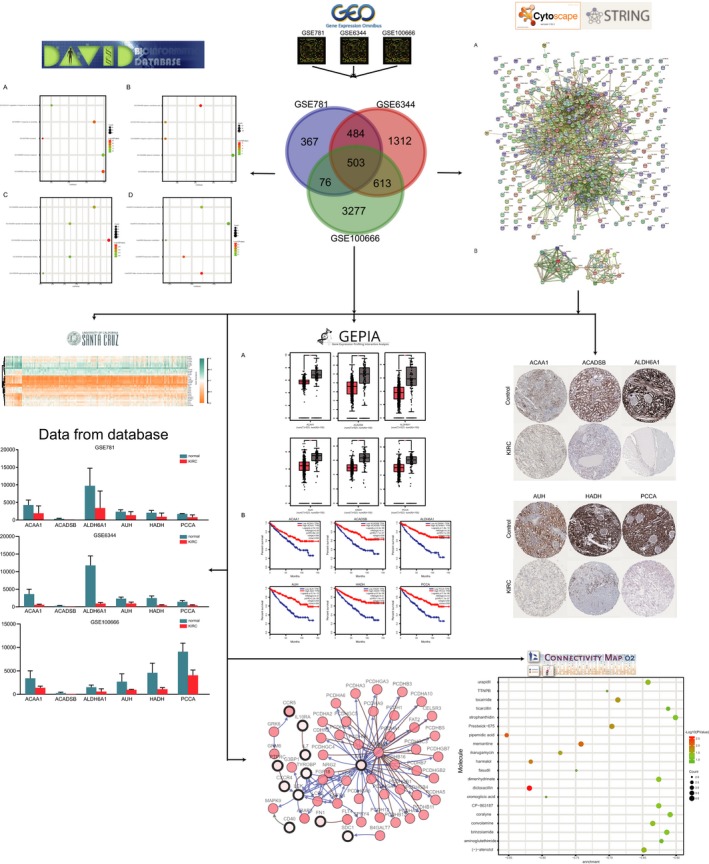
The workflow of this study for identifying key genes and pathways in KIRC

Renal cell carcinoma (RCC), also known as renal cell adenocarcinoma, accounts for more than 90% of primary renal neoplasms and pelvic cancer and ranks among the top 10 malignant tumors in both men and women. The incidence and mortality of RCC have been increasing over the past two decades. (Bray et al., 2018; Escudier et al., 2016; Hsieh, Le, Cao, Cheng, & Creighton, 2018; Srigley et al., 2013) Kidney renal clear cell carcinoma (KIRC) is the most common subtype of RCC (70%–75%) and represents one of the most malignant genitourinary cancers. Nearly a quarter of patients were diagnosed with distal metastasis and advanced regional disease. Although a major breakthrough has been made in the molecular mechanisms and therapeutic strategies for KIRC, the long‐term overall survival of patients has not been significantly improved, especially in patients with metastatic disease, with 5‐year survival <32%.(Linehan, 2012; Shuch et al., 2015) Therefore, identifying the promising novel diagnostic and prognostic biomarkers is urgently demanded, which will contribute to enhance our understanding of KIRC initiation and progression and promote early detection of KIRC. A previous study showed that Polybromo‐1 plays an important role in the proliferation and metastasis of KIRC by regulating multiple metabolic pathways including PI3K signaling pathway, glucose uptake pathway, and hypoxia response genes.(Chowdhury et al., 2016) In the past decade, with the rapid development of microarray and RNA‐sequencing technology, more and more biomarkers of tumor initiation, progression, and prognosis have been identified using bioinformatics analysis.(Sun et al., 2019; Yan et al., 2019) In this study, we integrated three microarray datasets from gene expression omnibus (GEO) database (GSE781, GSE6344, and GSE100666) to identify the differentially expressed genes (DEGs) between KIRC and adjacent normal tissues, aiming to explore and determine the promising novel biomarkers associated with pathogenesis and prognosis of KIRC. Meanwhile, we revealed some candidate small molecule drugs that could reverse the gene expression of KIRC based on the CMap database. The present study uncovered six novel biomarkers that were of great value in the diagnosis and prognosis of KIRC patients, which may contribute in revealing the molecular mechanisms of KIRC occurrence and progression. Figure 1 showed the workflow of our study.

## 2 MATERIALS AND METHODS

### 2.1 Data resources

We conducted a systematic search of GEO database for relevant microarray datasets in order to investigate the differential gene expression between KIRC and normal samples. GEO database (http://www.ncbi.nlm.nih.gov/geo/) is widely used to archive high‐throughput microarray experimental data, original series, and platform records. In total, the gene expression profiles of GSE781 (Lenburg et al., 2003), GSE6344 (Gumz et al., 2007; Tun et al., 2010), and GSE100666 were downloaded from GEO database. These RNA profiles were provided on GPL96 (Affymetrix Human Genome U133A Array) and platforms GPL16951 (Phalanx Human OneArray Ver. 6 Release 1).

### 2.2 Identification of DEGs

The original CEL files were downloaded and classified as KIRC and normal groups. The affy package of Bioconductor (http://www.bioconductor.org/) was used for data standardization and transforming raw data into expression values. The significance analysis of the empirical Bayes methods within limma package was applied to identify DEGs between KIRC samples and normal tissue sample.(Ritchie et al., 2015) The genes with the following cutoff criteria were considered as the significant DEGs: *p* < 0.05 and |logFC|>1.

### 2.3 Functional enrichment analysis

We performed gene ontology (GO) enrichment analysis for these overlap DEGs. GO enrichment analysis is a common useful method to analyze biological process (BP), molecular functions (MF), and cellular components (CC) of genes and gene products. KEGG pathway enrichment analysis was used to identify the potential functional and metabolic pathways associated with overlap DEGs. KEGG is a collection of databases that store a large number of information about genomes, biological pathways, diseases, chemical substances, and drugs. We performed GO enrichment and KEGG pathway analysis by using DAVID (Database for Annotation Visualization and Integrated Discovery). DAVID is a commonly used online biological information database that provides comprehensive functional annotation and pathway interpretations (version 6.7)(http://david.ncifcrf.gov).(Ashburner et al., 2000; Dennis et al., 2003; Gene Ontology Consortium, 2006; Huang da, Sherman, & Lempicki, 2009; Papanicolaou, Gebauer‐Jung, Blaxter, Owen McMillan, & Jiggins, 2008) *p* < 0.05 was considered statistically significant.

### 2.4 Protein–protein interaction (PPI) network construction and module analysis

The Search Tool for the Retrieval of Interacting Genes database (STRING, https://string-db.org/) was used to construct the PPI network.(Szklarczyk et al., 2015) The interactions with a combined score >0.4 were considered significant. The PPI network was visualized through the Cytoscape software. (Smoot, Ono, Ruscheinski, Wang, & Ideker, 2011) Subsequently, Molecular Complex Detection (MCODE) plug‐in of the Cytoscape software was applied to screen significant modules from the PPI network. The degree cutoff = 10, node score cutoff = 0.2, k‐core = 2, and max. depth = 100 were used as selection criterion.(Bandettini et al., 2012) We also performed Go and KEGG pathway enrichment analysis for the most significant modules. In addition, the Networks Gene Oncology tool (BiNGO) plugin in Cytoscape was used to perform and visualize the biological process analysis of the hub genes.(Maere, Heymans, & Kuiper, 2005)

### 2.5 Analysis and validation of hub genes

The hierarchical clustering of module genes was constructed by UCSC Cancer Genomics Browser (http://genome-cancer.ucsc.edu). A network of hub genes and their coexpression genes was established by cBioPortal online platform (http://www.cbioportal.org). The Gene Expression Profiling Interactive Analysis (GEPIA) was used to further confirm the reliability of the expression level of hub genes and their prognostic value.(Cerami et al., 2012; Gao et al., 2013; Tang et al., 2017) The hazard ratio (HR) with 95% confidence intervals (CI) of overall survival was calculated. The Kaplan–Meier curve and boxplot were visualized to present these relationships between gene expression and patients’ prognosis. The human protein atlas (HPA, www.proteinatlas.org) database was used to determine the protein expression of the hub genes between KIRC and normal tissues based on immunohistochemistry.

### 2.6 Identification of small molecules

We compared the overlap DEGs with those in the Connectivity Map database (CMap, http://www.broadinstitute.org/cmap/) to predict potential small active molecular that could reverse the current biological status of KIRC.(Lamb et al., 2006) First, the overlap DEGs were divided into upregulated and downregulated groups. Then different expression significant probesets from each group were selected for gene set enrichment analysis. Finally, the enrichment scores ranging from −1 to +1 were calculated. A positive connectivity value (closer to +1) indicated that the corresponding small molecules could induce the gene expression of KIRC, whereas a negative connectivity value (closer to −1) indicated greater similarity among the genes and the small molecules that could reverse the KIRC cell status.

## 3 RESULTS

### 3.1 Identification of DEGs in KIRC

**Figure 2:**
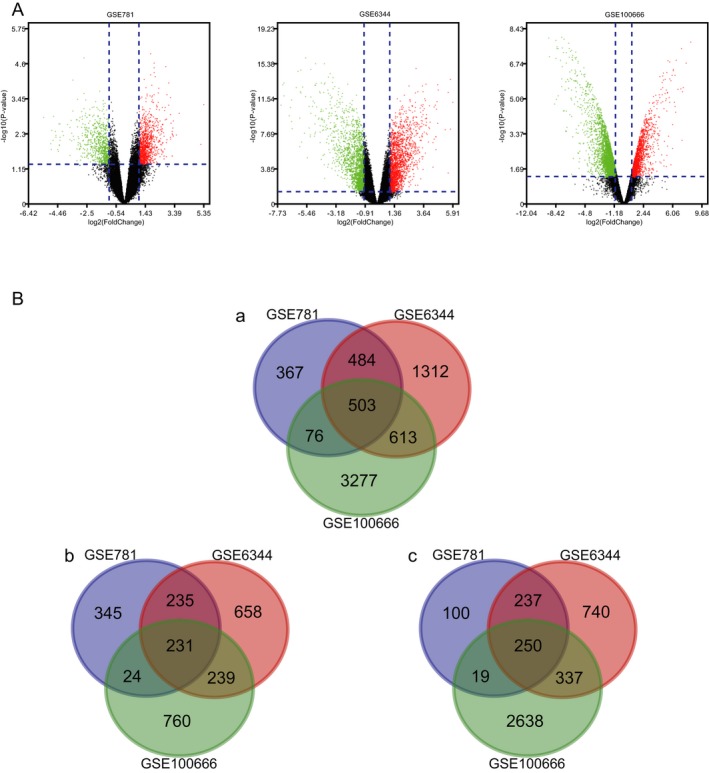
(A) Volcano plot of gene expression profile data between KIRC and normal tissues in each dataset. Red dots: significantly upregulated genes in KIRC; Green dots: significantly downregulated genes in KIRC; Black dots: nondifferentially expressed genes. *p* < 0.05 and |log2 FC|>1 were considered as significant. (B) a. Venn diagram of 503 overlap DEGs from GSE781, GSE6344, and GSE100666 datasets. b. Upregulated overlap DEGs; c. Downregulated overlap DEGs

After the standardization of GSE781, GSE6344, and GSE100666 datasets, a total of 503 overlap DEGs expressed in KIRC tissues were identified with the Limma package. The volcano plot of DEGs of KIRC in each dataset was presented in Figure 2A. The Venn diagrams showed the 503 overlap DEGs among the three datasets (Figure 2Ba), consisting of 231 upregulated genes (Figure 2Bb) and 250 downregulated genes (Figure 2Bc).

### 3.2 Enrichment analyses

**Figure 3:**
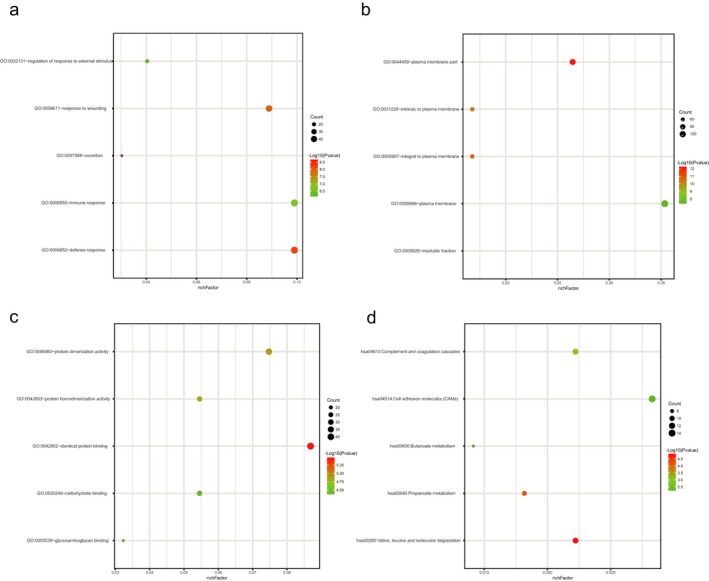
Functional and signaling pathway analysis of the overlapped DEGs in KIRC. (a) Biological processes (b) Cellular components (c) Molecular function (d) KEGG pathway

To better understand the biological functions of overlap DEGs among the three datasets, we performed GO function and KEGG pathway enrichment analysis using DAVID. For biological processes, GO analysis results indicated that upregulated and downregulated DEGs were significantly enriched in excretion, defense response, response to wounding, immune response, and regulation of response to external stimulus. Cell component analysis showed that these DEGs were particularly involved in plasma membrane part, integral to plasma membrane, intrinsic to plasma membrane, plasma membrane and insoluble fraction. Similarly, changes in molecular function of DEGs were significantly enriched in identical protein binding, protein dimerization activity, protein homodimerization activity, glycosaminoglycan binding, and carbohydrate binding. Additionally, the results of KEGG pathway analysis revealed that these DEGs were mainly enriched in valine, leucine, and isoleucine degradation, propanoate metabolism, complement and coagulation cascades, butanoate metabolism, and cell adhesion molecules (CAMs). (Figure 3;Table 1).

**Table 1:** Functional and pathway enrichment analysis of the overlap DEGs

Category	Term	*p* value
GOTERM_BP_FAT	GO:0007588~excretion	2.32E−09
GOTERM_BP_FAT	GO:0006952~defense response	6.27E−09
GOTERM_BP_FAT	GO:0009611~response to wounding	1.32E−08
GOTERM_BP_FAT	GO:0006955~immune response	2.22E−07
GOTERM_BP_FAT	GO:0032101~regulation of response to external stimulus	6.55E−07
GOTERM_CC_FAT	GO:0044459~plasma membrane part	9.91E−13
GOTERM_CC_FAT	GO:0005887~integral to plasma membrane	3.18E−11
GOTERM_CC_FAT	GO:0031226~intrinsic to plasma membrane	9.96E−11
GOTERM_CC_FAT	GO:0005886~plasma membrane	5.00E−08
GOTERM_CC_FAT	GO:0005626~insoluble fraction	2.16E−07
GOTERM_MF_FAT	GO:0042802~identical protein binding	3.31E−06
GOTERM_MF_FAT	GO:0,046,983 ~ protein dimerization activity	1.33E−05
GOTERM_MF_FAT	GO:0042803~protein homodimerization activity	1.54E−05
GOTERM_MF_FAT	GO:0005539~glycosaminoglycan binding	2.85E−05
GOTERM_MF_FAT	GO:0030246~carbohydrate binding	4.20E−05
KEGG_PATHWAY	hsa00280:Valine, leucine and isoleucine degradation	1.49E−05
KEGG_PATHWAY	hsa00640:Propanoate metabolism	5.26E−05
KEGG_PATHWAY	hsa04610:Complement and coagulation cascades	7.77E−04
KEGG_PATHWAY	hsa00650:Butanoate metabolism	0.003257
KEGG_PATHWAY	hsa04514:Cell adhesion molecules (CAMs)	0.005074

### 3.3 PPI network construction and module analysis

**Figure 4:**
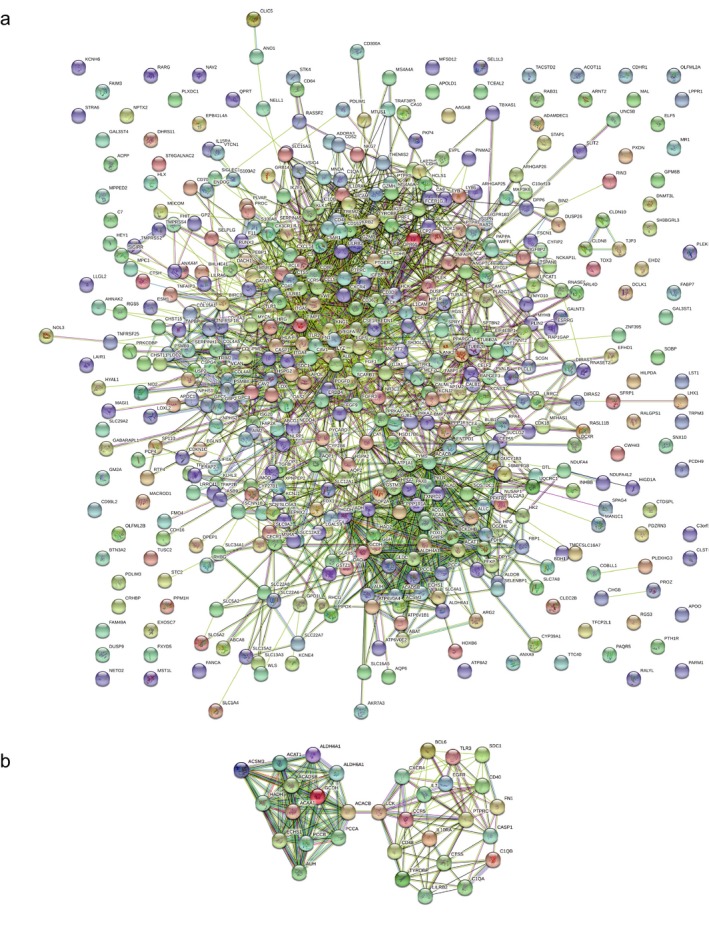
Protein–protein interaction networks construction and module analysis

**Figure 5:**
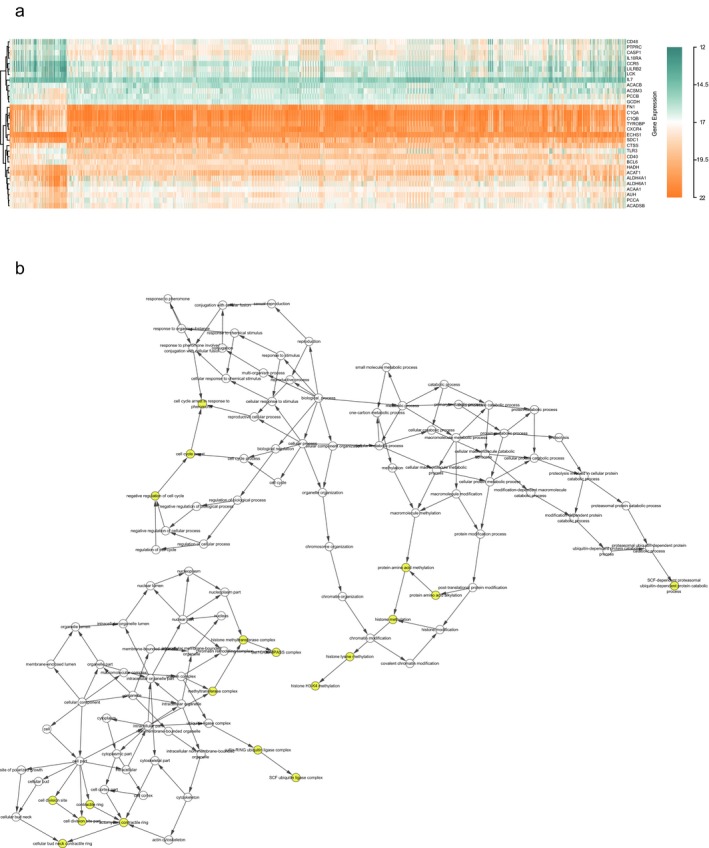
(a) The heatmap of module genes between KIRC and normal samples. (b) The biological process of module genes analyzed by BiNGO. The color depth of nodes represents the corrected *p*‐value. The size of nodes represents the number of genes involved

**Figure 6:**
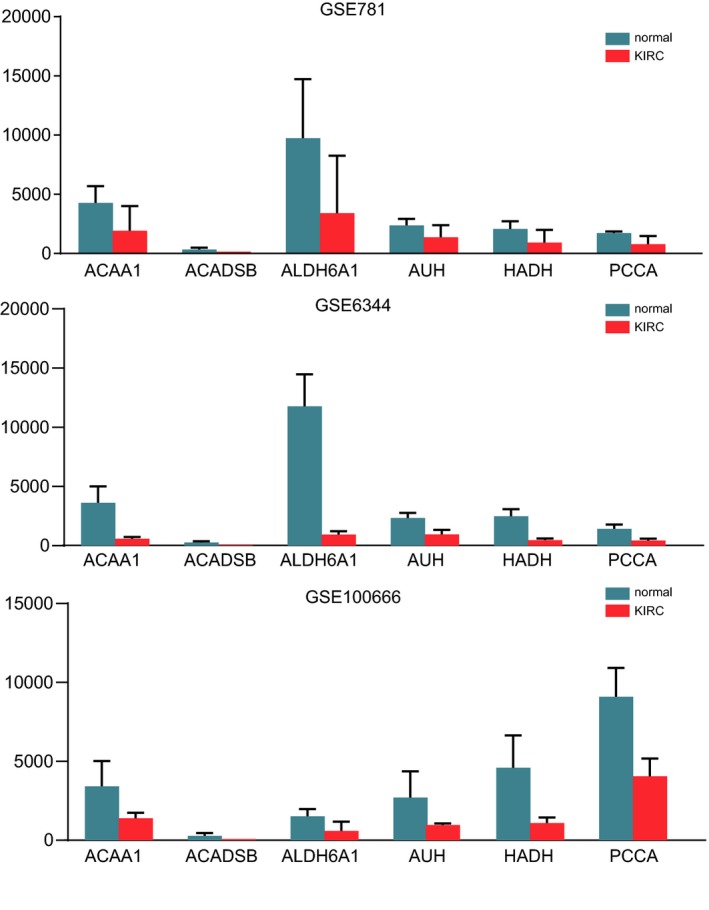
The expression level of hub genes between KIRC and normal tissues in three datasets

The PPI network among the DEGs was established by using the STRING database, with 417 nodes and 1912 interactions.(Figure 4a) Figure 4b presented the most significant module extracted from this PPI network by MCODE. Using KEGG pathway enrichment analysis, the genes in this module were mainly enriched in valine, leucine and isoleucine degradation, propanoate metabolism, and fatty acid degradation.(Table 2) Hierarchical clustering indicated that the module genes could basically differentiate the KIRC tissues from the noncancerous tissues.(Figure 5a) The biological process analysis indicated that the module genes were significantly related to cell cycle arrest, negative regulation of cell cycle, and cell cycle arrest in response to pheromone.(Figure 5b) *ACAA1, ACADSB, ALDH6A1, AUH, HADH,* *and PCCA* with high degree of connectivity were selected as hub genes. The expression level of *ACAA1, ACADSB, ALDH6A1, AUH, HADH,* *and PCCA* in KIRC tissues was significantly downregulated among each dataset.(Figure 6). Table S1 showed the full names and function roles of these hub genes.

**Table 2:** Functional and pathway enrichment analysis of genes in the most significant modules

ID	Pathway description	Observed gene count	False discovery rate
hsa280	Valine, leucine and isoleucine degradation	9	3.19E−15
hsa640	Propanoate metabolism	6	1.13E−09
hsa71	Fatty acid degradation	6	4.99E−09
hsa1212	Fatty acid metabolism	6	7.49E−09
hsa1100	Metabolic pathways	13	4.30E−07
hsa650	Butanoate metabolism	4	3.44E−06
hsa380	Tryptophan metabolism	4	1.60E−05
hsa1200	Carbon metabolism	5	1.81E−05
hsa310	Lysine degradation	4	3.44E−05
hsa4060	Cytokine–cytokine receptor interaction	6	7.84E−05

### 3.4 Analysis and validation of hub genes

**Figure 7:**
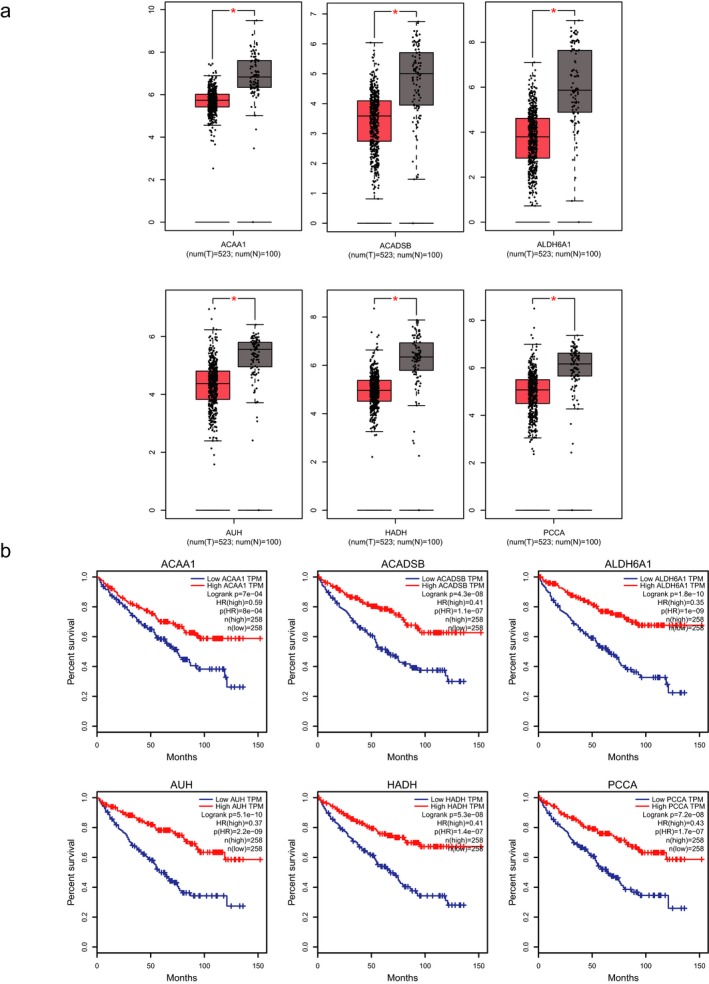
(a) The expression of hub genes between KIRC tissues and normal tissues. (b) The prognostic value of hub genes

**Figure 8:**
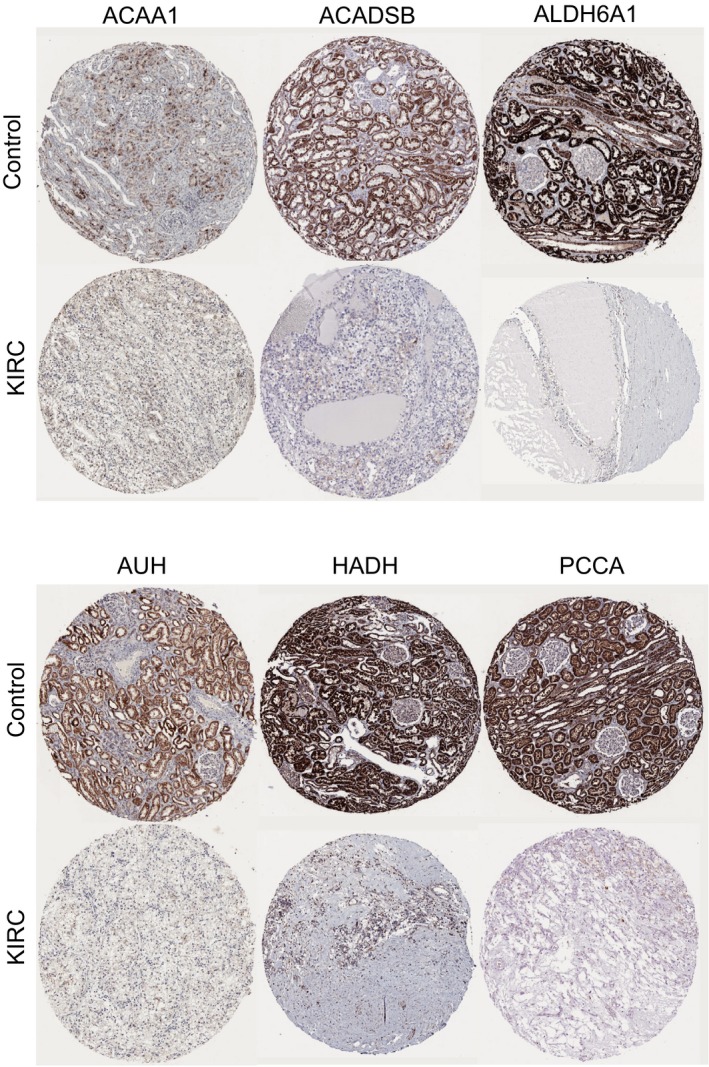
Representative immunohistochemistry staining results reveal the protein level expression of hub genes in KIRC and normal tissues

**Figure 9:**
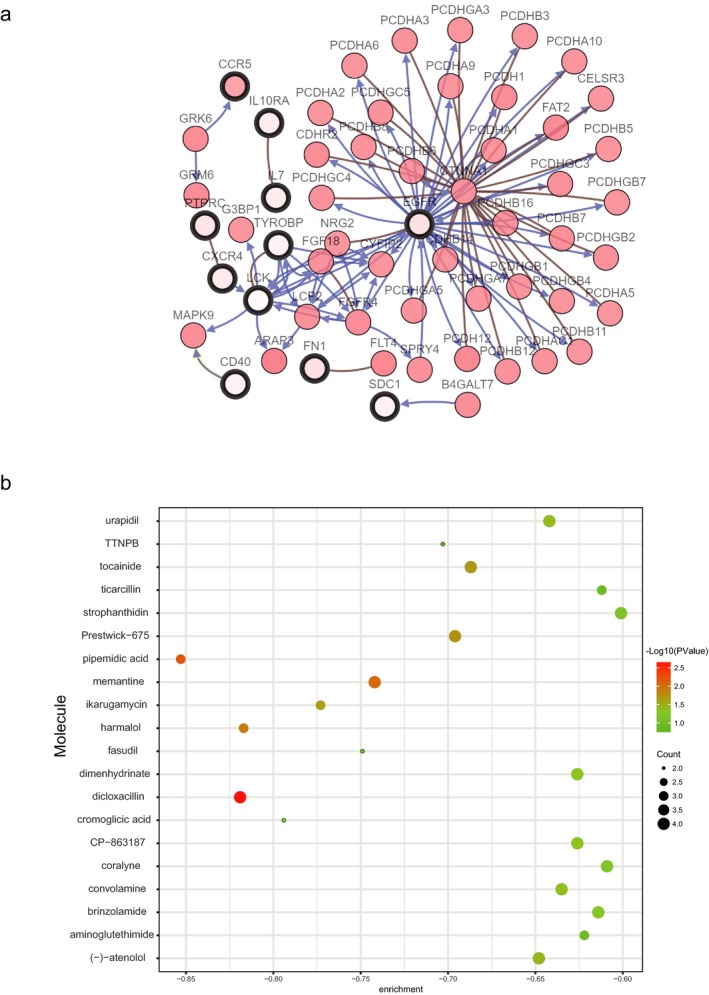
(a) The network of module genes and their coexpression genes constructed by cBioPortal. Nodes with thick outline: hub genes; Nodes with thin outline: coexpression genes. (b) Pop plot of top 20 identified small molecules that could reverse the gene expression of KIRC

The hub gene expression and their prognostic correlation were further analyzed by GEPIA database. The mining of GEPIA database further confirmed that these hub genes were significantly downregulated in KIRC tissues compared to normal tissues.(Figure 7a) Based on the prognostic analysis of 516 KIRC patients in GEPIA database, we found that downregulation of *ACAA1, ACADSB, ALDH6A1, AUH, HADH,* *and PCCA* were correlated with significantly worse overall survival of patients.(Figure 7b) The expression level of these hub genes could represent the important prognostic biomarkers for predicting the survival of KIRC patients. Due to the gene expression not always consistent with its protein content, (Maier, Guell, & Serrano, 2009) we used HPA database to further analyze the protein level of hub genes. The immunohistochemical staining results indicated significantly higher positivity for *ACAA1, ACADSB, ALDH6A1, AUH, HADH,* *and PCCA* in adjacent normal tissues than in cancer tissues.(Figure 8) A network of the module genes and their coexpression genes was constructed using cBioPortal online platform.(Figure 9a) To further explore the molecular mechanism of the hub genes in KIRC, we predicted potential transcription factors and constructed a regulatory network of lncRNA, miRNA, and mRNA which could be involved in the expression of hub genes by GGBI analysis.(Gene‐Cloud Biotechnology Information, Figure S1 & S2).

### 3.5 Identification of related active small molecules

In order to screen and identify potential therapeutic drugs for KIRC, we uploaded these DEGs which were divided into upregulated and downregulated groups into the CMap database and then matched them with small molecule treatment. The top 20 most significant small molecules with their enrichment scores were shown in Table 3 and Figure 9b. The small molecules of pipemidic acid (enrichment score = −0.853) and dicloxacillin (enrichment score = −0.819) were related to highly significant negative scores, which could imitate the status of normal cells. These potential small molecule drugs could reverse the gene expression induced by KIRC, thus providing new directions and molecular mechanisms for the future development of new targeted drugs for treating KIRC. However, further studies were still required to confirm the value of these candidate small molecules in treating KIRC.

**Table 3:** List of the 20 most significant small molecule drugs that can reverse the tumoral status of KIRC

CMap name	Enrichment	*p*
Pipemidic acid	−0.853	0.00637
Dicloxacillin	−0.819	0.00203
Harmalol	−0.817	0.01216
Cromoglicic acid	−0.794	0.08303
Ikarugamycin	−0.773	0.024
Fasudil	−0.749	0.12454
Memantine	−0.742	0.00871
TTNPB	−0.703	0.17597
Prestwick−675	−0.696	0.01814
Tocainide	−0.687	0.02085
(‐)‐Atenolol	−0.648	0.03648
Urapidil	−0.642	0.03957
Convolamine	−0.635	0.04323
Dimenhydrinate	−0.626	0.04812
CP−863187	−0.626	0.04846
Aminoglutethimide	−0.622	0.11319
Brinzolamide	−0.614	0.05602
Ticarcillin	−0.612	0.12627
Coralyne	−0.609	0.05954
Strophanthidin	−0.601	0.06595

## 4 DISCUSSION

KIRC is the most lethal urological disease and the incidence has increased over the past few decades. KIRC is also a heterogeneous histological disease and the mechanisms underlying its occurrence and progression are complex. (Van Poppel et al., 2011; Xue et al., 2012) Therefore, identifying the key genes related to the pathogenesis and prognosis of KIRC is urgently demanded. In the present study, we integrated gene expression profiles of GSE781, GSE6344, and GSE100666 from GEO database to screen the differentially expressed genes between KIRC and adjacent normal tissues by bioinformatics techniques. The purpose of our study was to find the novel diagnostic and prognostic biomarkers for KIRC. Additionally, we also identified the candidate small molecule drugs that could reverse the tumoral status of KIRC in order to provide new directions for the future development of new drugs for treating KIRC.

A total of 503 overlap DEGs were found among the three datasets, including 231 significantly upregulated genes and 250 downregulated genes. We analyzed the biological process, molecular function, and cellular component of these overlap DEGs using GO enrichment analysis in order to explore their role in the occurrence and development of KIRC. Excretion, defense response, and response to wounding were the top three significantly major functions among the biological process. Molecular function enriched for the DEGs were mainly within identical protein binding, protein dimerization activity, and protein homodimerization activity. Changes in cell component were mainly associated with plasma membrane part, integral to plasma membrane, and intrinsic to plasma membrane. Furthermore, the results of KEGG pathway suggested these DEGs were significantly enriched in valine, leucine and isoleucine degradation, propanoate metabolism, complement and coagulation cascades, butanoate metabolism, and cell adhesion molecules. The cell adhesion molecules are widely involved in the intercellular and cellular–extracellular matrix interactions of tumors. Some adhesion molecules play an important role in tumor recurrence, metastasis, and invasion.(Okegawa, Pong, Li, & Hsieh, 2004) Based on the construction of PPI network and module analysis, *ACAA1, ACADSB, ALDH6A1, AUH, HADH,* *and PCCA* with high degree of connectivity were selected as hub genes. They were significantly downregulated in KIRC tissues compared with normal tissues. We used GEPIA database to further validate the results of bioinformatics analysis. Similarly, the mining of GEPIA database also confirmed that these hub genes exhibited significant differences in expression between KIRC and normal tissues. More importantly, these hub genes have important value in predicting the survival of KIRC patients. The low expression level of *ACAA1, ACADSB, ALDH6A1, AUH, HADH,* *and PCCA* was significantly correlated with the prognosis of patients. The immunohistochemical staining results revealed that the expression level of hub gene was consistent with their protein expression level, which further validated the accuracy of our findings. The present study first revealed six key genes with the diagnostic, prognostic, and therapeutic value in KIRC. No studies have reported the role of these six key genes in the initiation and progression of KIRC. In order to further enhance the understanding of these hub genes in the pathogenesis of KIRC, we predicted the corresponding potential transcription factors and establish a regulatory network of lncRNA–miRNA–mRNA for each hub gene. The construction of these regulatory networks will contribute in revealing the potential mechanism of these hub genes in KIRC initiation and progression. *CENPK*, a member of the centromeric protein family, was markedly upregulated in ovarian cancer tissues and associated with poor prognosis in patients. *ALDH6A1* and *ALDH6A1* were significantly upregulated in metastatic prostate cancer and could be used to predict the survival of patients.(Cho et al., 2018) A previous study showed that *HADH* knockdown can promote tumor cell migration and invasion by activing Akt signaling pathway.(Shen et al., 2017) Evidence indicated that *ACADSB* was also markedly downregulated in HCC cell lines.(Nwosu et al., 2018) However, no studies have reported the effects of hub genes on the initiation and progression of KIRC. The above studies indicated these hub genes could play an important role in the occurrence and development of KIRC.

In addition, we used the CMap database to determine a set of potential small molecules which may imitate the gene expression of normal cells. The small molecules with higher negative enrichment scores meant that they are more likely to reverse KIRC gene expression. This work will contribute to developing new target therapeutic drugs for KIRC. The efficacy and safety of pipemidic acid, the most significant small molecule (enrichment score = −0.853), has not been investigated as yet in KIRC. Meanwhile, the relationship between dicloxacillin (enrichment score = −0.819) and KIRC remains little known. Therefore, further studies were urgently demanded to reveal the huge potential of these listed small molecules in treating KIRC.

In conclusion, through a comprehensive bioinformatics analysis for the microarray datasets of KIRC, we first uncovered six novel key genes associated with KIRC initiation and progression. These hub genes could serve as promising novel biomarkers for the diagnosis, prognosis, and treatment of KIRC. A series of detailed analysis were further confirmed their vital roles on the pathogenesis of KIRC. In addition, this study identified a group of candidate small molecule drugs, which could provide new directions for the development of new targeted drugs for KIRC. Taken together, this work provided new insights for KIRC to integrate multiple biomarkers in clinical practices.

## CONFLICTS OF INTERESTS

None.
