# Supplementary material for: The promising novel biomarkers and candidate small molecule drugs in kidney renal clear cell carcinoma: Evidence from bioinformatics analysis of high‐throughput data
Source: Mol Genet Genomic Med. 2019 Feb 21;7(5):e607. doi: 10.1002/mgg3.607 (PMC6503072; doi:10.1002/mgg3.607)
Supplement: Supplementary file 3 [file MGG3-7-e607-s003.docx]

**Table S1** The full name and functional roles of eight hub genes.

| Gene symbol | Full name | Funcution |
| --- | --- | --- |
| ACAA1 | Acetyl-CoA Acyltransferase 1 | This gene encodes an enzyme operative in the beta-oxidation system of the peroxisomes. Deficiency of this enzyme leads to pseudo-Zellweger syndrome. Alternative splicing results in multiple transcript variants. |
| ACADSB | Acyl-CoA Dehydrogenase Short/Branched Chain | Short/branched chain acyl-CoA dehydrogenase(ACADSB) is a member of the acyl-CoA dehydrogenase family of enzymes that catalyze the dehydrogenation of acyl-CoA derivatives in the metabolism of fatty acids or branch chained amino acids. Substrate specificity is the primary characteristic used to define members of this gene family. The ACADSB gene product has the greatest activity towards the short branched chain acyl-CoA derivative, (S)-2-methylbutyryl-CoA, but also reacts significantly with other 2-methyl branched chain substrates and with short straight chain acyl-CoAs. The cDNA encodes for a mitochondrial precursor protein which is cleaved upon mitochondrial import and predicted to yield a mature peptide of approximately 43.7-KDa. |
| ALDH6A1 | Aldehyde Dehydrogenase 6 Family Member A1 | This gene encodes a member of the aldehyde dehydrogenase protein family. The encoded protein is a mitochondrial methylmalonate semialdehyde dehydrogenase that plays a role in the valine and pyrimidine catabolic pathways. This protein catalyzes the irreversible oxidative decarboxylation of malonate and methylmalonate semialdehydes to acetyl- and propionyl-CoA. Methylmalonate semialdehyde dehydrogenase deficiency is characterized by elevated beta-alanine, 3-hydroxypropionic acid, and both isomers of 3-amino and 3-hydroxyisobutyric acids in urine organic acids. Alternate splicing results in multiple transcript variants. |
| AUH | AU RNA Binding Methylglutaconyl-CoA Hydratase | This gene encodes bifunctional mitochondrial protein that has both RNA-binding and hydratase activities. The encoded protein is a methylglutaconyl-CoA hydratase that catalyzes the hydration of 3-methylglutaconyl-CoA to 3-hydroxy-3-methyl-glutaryl-CoA, a critical step in the leucine degradation pathway. This protein also binds AU-rich elements (AREs) found in the 3' UTRs of rapidly decaying mRNAs including c-fos, c-myc and granulocyte/ macrophage colony stimulating factor. ARE elements are involved in directing RNA to rapid degradation and deadenylation. This protein is localizes to the mitochondrial matrix and the inner mitochondrial membrane and may be involved in mitochondrial protein synthesis. Mutations in this gene are the cause of 3-methylglutaconic aciduria, type I. Alternative splicing results in multiple transcript variants. |
| HADH | Hydroxyacyl-CoA Dehydrogenase | This gene is a member of the 3-hydroxyacyl-CoA dehydrogenase gene family. The encoded protein functions in the mitochondrial matrix to catalyze the oxidation of straight-chain 3-hydroxyacyl-CoAs as part of the beta-oxidation pathway. Its enzymatic activity is highest with medium-chain-length fatty acids. Mutations in this gene cause one form of familial hyperinsulinemic hypoglycemia. The human genome contains a related pseudogene of this gene on chromosome 15. |
| PCCA | Propionyl-CoA Carboxylase Subunit Alpha | The protein encoded by this gene is the alpha subunit of the heterodimeric mitochondrial enzyme Propionyl-CoA carboxylase. PCCA encodes the biotin-binding region of this enzyme. Mutations in either PCCA or PCCB (encoding the beta subunit) lead to an enzyme deficiency resulting in propionic acidemia. Multiple transcript variants encoding different isoforms have been found for this gene. |
